# Evaluation of strategies for the occupational health risk assessment of chemical toxicants in the workplace based on a quantitative analysis model

**DOI:** 10.3389/fpubh.2022.1035065

**Published:** 2022-11-16

**Authors:** Qiuliang Xu, Meibian Zhang, Lingtong Xu, Weiming Yuan, Hong Ren, Peng Wang, Xincun Shao, Zhen Zhou, Hua Zou, Yiyao Cao

**Affiliations:** ^1^Zhejiang Provincial Center for Disease Control and Prevention, Hangzhou, China; ^2^Chinese Center for Disease Control and Prevention, National Institute of Occupational Health and Poison Control, Beijing, China; ^3^Zhejiang Tianlan Environmental Protection Engineering Co., Ltd., Hangzhou, China; ^4^Zhejiang Jidi Testing Technology Co., Ltd., Taizhou, China

**Keywords:** methodology, occupational health, risk assessment, workplace, chemical toxicants

## Abstract

**Objectives:**

The commonly used methods for the occupational health risk assessment (OHRA) of chemical toxicants cannot fully meet the needs of practical work. This study evaluated OHRA strategies for chemical toxicants in the workplace by establishing a quantitative analysis model.

**Methods:**

Five typical industries in China that implement OHRA using the six common models (the Environmental Protection Agency, Australian, Romanian, Singaporean, International Council on Mining and Metals, and the Control of Substances Hazardous to Health models) were selected as the research objects. We established a quantitative analysis model to compare the six models and applied it to compare the results obtained using each model and preliminarily analyze the advantages, limitations, and application scope of each method.

**Results:**

The risk ratio (RR) values of the six methods decreased in the following order: RR_EPA_ > RR_COSHH_ > RR_ICMM_ > RR_Australia_ > RR_Singaporean_ > RR_Romanian_ (*P* < 0.05). Among the six models, the Singaporean model had the strongest RR correlation with the other models (*P* < 0.01). The sequence of RRs obtained from the Singaporean, ICMM, Australian, and Romanian models in the five industries was consistent with the sequence of the three inherent risk levels in those industries. Only the Romanian model could distinguish between the RRs of all five industries. The EPA and Singaporean models could effectively distinguish the differences in inherent risk for four hazard factors (manganese and inorganic compounds, benzene, xylene, and ethyl acetate), with the assessment accuracy being relatively higher for the EPA model.

**Conclusions:**

Among the six models, the EPA model had the relatively highest accuracy in assessing chemical toxicants, followed by the Singaporean model. The EPA and Romanian models were strongest in differentiating the differences in toxicity risk. More studies on OHRA methodology are needed.

## Introduction

Occupational injuries caused by exposure to chemical toxicants are serious problems worldwide. Globally, air pollution-related diseases kill approximately seven million people each year (1). Over 140 million chemicals are registered in the International Chemical Abstracts Society, and ~10,000 new chemicals are registered each year (2), making chemical-related occupational injuries complicated. Occupational chemical poisoning has a high fatality rate and can easily result in a public health emergency (3).

At present, the common methods for assessing chemical hazards in the workplace are based on occupational exposure limits (OELs) and threshold limit values (TLVs) from the American conference of governmental industrial hygienists (ACGIH). However, the ACGIH values include only a few hundred chemicals, and values are not available for most chemical toxicants. These methods also depend on the concentration of chemical toxicants in the workplace, which creates problems in cases where the on-site concentrations of chemical toxicants cannot be obtained. Moreover, new chemicals are introduced in industry and commerce much faster than new occupational exposure limits can be established. Due to these technical limitations, assessment methods based on OELs and TLVs are unable to meet the actual work requirements.

Occupational health risk assessment (OHRA) is a comprehensive and systematic identification and analysis of workplace hazards based on the identification and analysis of hazard factors and protective measures in the workplace. OHRA provides a quantitative assessment of the level of occupational health risk that can inform corresponding control measures to supplement existing prevention and control strategies for occupational diseases. In 1983, the National Research Council of the United States first proposed the theory of risk assessment, which divided OHRA into four stages: hazard identification, dose-response relationship assessment, exposure assessment, and risk characterization (4). Since then, various OHRA methods have been promulgated by European and American agencies and international organizations. At present, over 10 OHRA methods are employed worldwide, including qualitative, quantitative, and semi-quantitative methods; among them, the following six are the most common: the United States Environmental Protection Agency (EPA) model (5), the United Kingdom's Control of Substances Hazardous to Health Essentials (COSHH) model (6), the Singaporean model (7), the International Council on Mining and Metals (ICMM) model (8), the Australian model (9), and the Romanian model (10). Based on the EPA, Singaporean, and COSHH models, China launched technical guidelines for the OHRA of chemicals in the workplace (GBZ/T 298-2017).

The OHRA methods have different principles and methodological characteristics due to their different backgrounds, national conditions, and initial fields of application, resulting in model-specific advantages and limitations (11, 12). Little research has been conducted to understand the differences among methods and develop guidance on the appropriate assessment methods for specific poisons or sites. This is because we do not fully understand the theoretical underpinnings, scope of applicability, and classification parameters of the degree of hazard for each method.

To identify a suitable OHRA strategy for chemical toxicants in the workplace, we have studied OHRA methods for nearly 10 years. Through a literature review, we qualitatively compared six commonly used OHRA methods (EPA, COSHH, Singaporean, ICMM, Australian, and Romanian), revealing the strengths and limitations of each method (13), which provide some guidance for our practical work, but they are still not precise enough. In order to evaluate the applicability of each method in practice guidance work, we applied six methods for risk assessment in typical industries in China. We found that using different methods to assess the same hazard often produces inconsistent results (14–19). In order to understand which method is relatively more reliable in assessing the risk of chemical toxicants, we introduced the concept of risk ratio (RR) to compare the assessment results of each method, and used various methods to verify the comparison results. We applied the methodology to over 70 enterprises in seven typical industries (e.g., wood furniture manufacturing, electroplating, crane manufacturing, printing and dyeing, printing, leather products manufacturing and mining) and found that the EPA and Singaporean models exhibited good reliability since they could distinguish the inherent risk of the industry or risk factor and tended to get higher risk levels (20–23). Through the above research, it can be seen that the quantitative comparison framework introduced by RR can be used as a method to evaluate the relative reliability of each method. And the framework that we've created is open, we can develop more reliable validation models and apply them in more and more extensive hazard sites to understand more differences among the models based on solve practical problems.

Chemical toxicants seriously endanger human health. OHRA is an effective way to control the occupational health risk of harmful toxicants in the case of inadequate standards and regulations. Understanding the differences between methods and the scope of application of each method is of great significance for guiding practical work. On the basis of previous research, we selected five typical industries in China (soil sand mining, ferrous metal casting, ship repair, equipment repair, and gasoline station) as the research objects and performed OHRA for exposure to chemical hazards using six OHRA methods (EPA, COSHH, Singaporean, ICMM, Australian, and Romanian). Using the established quantitative analysis model that improved on early-stage qualitative and quantitative analysis model, we discussed the correlation and accuracy of the evaluation results for each method along with the differences among methods. We also preliminarily analyzed the advantages, limitations, and application scope of each method. The results provide a scientific basis OHRA-based occupational health management in countries facing occupational hazards. The findings also provide valuable information for further application and methodological research on OHRA.

## Materials and methods

### Description of typical industries

Soil sand mining, ferrous metal casting, ship repair, equipment repair, and gasoline station were selected as typical industries for the following reasons. According to the “Management catalog of occupational hazard risk classification of construction projects” issued by the State Administration of Work Safety of China (2012 edition) (24), the inherent risk (IR) for occupational hazards in the soil sand mining and ferrous metal casting industries was classified as “severe.” The IR for the ship repair and equipment repair industries was classified as “medium,” while that for the gasoline station industry was classified as “low.” Thus, these five industries represent a range of IR levels in China (severe, medium, and low IR). Among the five industries, IR for occupational hazards decreases in the following order: IR_mining_ and IR_ferrous_ > IR_ship_, and IR_equipment_ > IR_gasoline_.

A total of 151 enterprises in Zhejiang Province in eastern China were selected as typical enterprises. These included three large enterprises, eight medium-sized enterprises, 29 small enterprises and 111 micro-enterprises (25). A total of ~16,000 workers exposed to hazard factors were involved. Basic information is shown in Table 1.

**Table 1:** General information and exposure levels of hazard factors in five typical industries.

**Industry (*n*)**	**Location**	**No. of** **locations**	**Hazard factor**	**Exposure levels** **by ratio** **(mean, range) (mg/m^3^)**	**Evaluation** **by China** **PC-TWA**	**Evaluation** **by ACGIH** **TLV-TWA**
Mining of soil and sand	Rig operator	19	Silicious dust	2.275 (0.186–21.6)	Disqualified	Disqualified
(12)	Excavator driver	26	Silicious dust	0.892 (0.143–2.986)	Disqualified	Disqualified
	Transport driver	25	Silicious dust	1.107 (0.200–3.429)	Disqualified	Disqualified
	Stope inspector	11	Silicious dust	0.989 (0.333–2.186)	Disqualified	Disqualified
	Discharge	15	Silicious dust	2.216 (0.357–10.729)	Disqualified	Disqualified
	Crushing inspector	26	Silicious dust	1.218 (0.186–4.714)	Disqualified	Disqualified
	Forklift driver	15	Silicious dust	0.901 (0.171–4.233)	Disqualified	Disqualified
	Sprinkler driver	11	Silicious dust	0.617 (0.143–0.943)	Qualified	Disqualified
Ferrous casting	Molding	43	Silicious dust	1.372 (0.200–7.200)	Disqualified	Disqualified
(17)	Smelting	6	Other dust	0.158 (0.050–0.363)	Qualified	/
	Casting	20	Silicious dust	0.761 (0.020–1.660)	Disqualified	Disqualified
			Other dust (iron)	0.136 (0.030–0.363)	Qualified	/
	Sand stripping	23	Silicious dust	1.237 (0.150–7.500)	Disqualified	Disqualified
	Shot blasting	5	Silicious dust	5.900 (0.500–13.60)	Disqualified	Disqualified
Ship repairs	Electrowelding	208	Welding fume	1.355 (0.050–7.575)	Disqualified	/
(11)			Manganese and inorganic compounds	0.956 (0.003–28.98)	Disqualified	Disqualified
			Nitrogen oxides	0.013 (0.002–0.038)	Qualified	Qualified
	Polishing	176	Grinding wheel dust	0.618 (0.025–5.378)	Disqualified	/
	Spraying	44	Benzene	0.037 (0.008–0.200)	Qualified	Qualified
			Xylene	1.149 (0.001–12.89)	Disqualified	Disqualified
			Ethyl acetate	0.002 (0.0003–0.031)	Qualified	Qualified
	Sanding	55	Iron-ore dust	1.549 (0.060–5.483)	Disqualified	Qualified
Equipment repair	Electrowelding	12	Welding fume	0.071 (0.025–0.225)	Qualified	/
(11)			Manganese and inorganic compounds	0.027 (0.007–0.073)	Qualified	Qualified
	Polishing	11	Grinding wheel dust	0.032 (0.014–0.074)	Qualified	/
	Paint mixing	3	Benzene	0.05	Qualified	Qualified
			Xylene	0.023 (0.010–0.030)	Qualified	Qualified
			Ethyl acetate	0.0007	Qualified	Qualified
	Spraying	9	Benzene	0.068 (0.008–0.1)	Qualified	Qualified
			Xylene	0.0468 (0.001–0.16)	Qualified	Qualified
			Ethyl acetate	0.001 (0.0004–0.005)	Qualified	Qualified
	Polishing	15	Talc dust	0.127 (0.025–0.525)	Qualified	Qualified
Petrol station	Oiling	100	Ggasoline	0.044 (0.0003–0.491)	Qualified	Qualified
(100)	Oil discharge	100	Gasoline	0.006 (0.0003–0.096)	Qualified	Qualified

ACGIH TLV-TWA, threshold limit values-time weighted average permitted by the American Conference of Governmental Industrial Hygienists; PC-TWA, permissible concentration-time weighted average.

### Identification and detection of hazard factors

The hazard factors and levels of exposure were identified through occupational health field investigations, air sampling, and laboratory testing. Air sampling and laboratory testing were carried out in accordance with the Chinese standard “Specifications of air sampling for hazardous substances monitoring in the workplace (GBZ 159)” and “Determination of toxic substances in workplace air (GBZ/T160 and 300).” Table 1 shows the basic information and levels of exposure to hazard factors (e.g., silicon dust, welding dust, manganese and inorganic compounds, grinding wheel dust, xylene, and iron ore powder) in each industry. The exposure levels of hazard factors at some locations in the soil sand mining, ferrous metal casting, and ship repair industries exceeded the permissible concentration-time weighted average (PC-TWA) permitted by China or the threshold limit values-time weighted average (TLV-TWA) permitted by ACGIH. This was not the case for the equipment repair and gasoline station industries.

### Introduction to the six commonly used OHRA methods

The six common OHRA methods (EPA, COSHH, Singaporean, ICMM, Australian, and Romanian) have similar assessment frameworks (22). The main assessment framework is based on the degree of hazard, exposure level, and probability of occurrence and includes hazard identification, hazard characteristic assessment, exposure assessment, and risk description. The detailed principles of the six methods have been reported previously (5–10) and are briefly described below.

(1) EPA method (quantitative evaluation). The EPA inhalation risk assessment consists of two parts: carcinogenic and non-carcinogenic risk assessments. The non-carcinogenic risk assessment was mainly applied in this study and involves two primary steps:
A) Estimating exposure concentration (EC, in μg/m^3^):
  (1) $$\begin{array}{l} \text{EC }=\;\left(\text{CA }\times \text{ ET }\times \text{ EF }\times \text{ ED}\right)/\text{AT} \end{array}$$
  where CA (μg/m^3^) is the concentration of hazard factor in the air; ET (h/d) is the exposure time; EF (days/year) is the exposure frequency; ED (years) is the exposure duration; AT [ED (years) × 365 days/year × 24 h/day] is the average exposure time.
B) Non-carcinogenic risk assessment:
  The hazard quotient (HQ), which indicates the risk level, is defined as
  (2) $$\begin{array}{l} \text{HQ }=\text{ EC}/\text{RfC }\times \;1,000\;\left(\text{μg}/\text{mg}\right) \end{array}$$
  where RfC (mg/m^3^) is the reference concentration of inhalation toxicity.

The EPA model can calculate the occupational health risk level of chemical toxicants with relative accuracy, but can only assess the health risk caused by inhalation route, and is limited to chemical toxicants with reference concentration (RfC) and inhalation unit risk (IUR), which can only be retrieved from the EPA website poison database.

(2) COSHH model for qualitative evaluation. In this method, the health hazard levels and exposure levels of chemical substances (solid or liquid) are considered comprehensively, and the control level is provided by a matrix method. The health hazard level of a chemical is determined according to a hazard band using risk phrases or OELs. The exposure level is determined according to the dustiness of a solid or the volatility of a liquid and the scale of use. While this method is simple and feasible, it may not always be accurate because it does not consider protection measures or on-site toxicant concentrations.
(3) Singaporean method (semi-quantitative evaluation). The risk level is calculated according to the hazard ratings (HR) and exposure ratings (ER), and the formula is as follows:
  (3) $$\begin{array}{l} \text{Risk }=\;{\left(\text{HR}\times \text{ER}\right)}^{1/2} \end{array}$$
  The HR is determined based on carcinogenicity classifications from the ACGIH and the International Agency for Research on Cancer, or on the acute toxicity data of chemicals (LD50 and LC50). The ER is classified according to the ratio of field exposure concentration to occupational exposure limits.
(4) ICMM method (qualitative evaluation). This method comprehensively considers the possible health hazards, probability of exposure, and exposure time. The risk level is determined using a quantitative method or matrix method.
(5) Australian method (qualitative evaluation). In this method, the risk levels are determined manually using a diagram or a calculator based on the likelihood of occurrence, frequency of exposure, and severity of consequences. This method is simple and easy to apply and is suitable for a wide range of assessments (e.g., risk assessments carried out by occupational health management personnel in small- and medium-sized enterprises (26).
(6) Romanian method (qualitative assessment). In this method, the risk level is evaluated using a matrix method based on the severity and probability of consequences resulting from hazard factors. This method can be used to calculate the overall risk level of the workplace and has obvious advantages in comprehensive risk assessment.

### Quantitative analysis model

#### Risk ratio (RR)

The six OHRA methods produced different levels of risk (22). To compare the results of each method, the risk levels obtained using the six methods were converted into RRs for quantitative comparison.

(1) Conversion of risk level: The EPA method produces quantitative data. The output of the COSHH method is control method classification. The risk assessment results of the other four methods are classifications of risk level. Thus, to compare the assessment results among different methods, the EPA non-carcinogenic risk assessment results (HQ) were converted into risk level by referring to the classification standard of exposure concentration of the Singaporean method, which includes five levels. The results of the COSHH method were converted by referring to the risk level of the Singaporean method (Table 2).
(2) RR calculation: After risk level conversion, the results for the six methods were converted to the classification of risk level. The risk assessment results of the EPA, Australian, Singaporean, and ICMM models were divided into five levels, while those of the Romanian and COSHH models were divided into seven and four levels, respectively. The concept of RR was introduced to allow comparison among the risk assessment results of different methods. RR was defined as the ratio of the risk level of an occupational hazard factor assessed by a method to the highest risk level of the model. The RR represents the relative risk level of hazard factors derived from a certain method.

**Table 2:** Conversion of risk assessment results for the EPA and COSHH models.

**The EPA model**		**The COSHH model**	
**Hazard** **quotient (HQ)**	**Risk** **level^†^**	**Control** **strategy**	**Risk** **level^‡^**
<0.1	1	–	–
0.1–0.5	2	CS1	2
0.5–1.0	3	CS2	3
1.0–2.0	4	CS3	4
≥2.0	5	CS4	5

† Modified based on the classification standard of exposure concentration of the Singaporean model.

‡ Modified based on the risk level of the Singaporean model.

#### Concentration ratio (CR)

To make the exposure concentration of hazard factors of different positions comparable, CR was defined as the ratio of the exposure concentration of a hazard factor to the OEL of the hazard factor (22). CR represents the relative exposure level of a certain hazard factor in a certain position; thus, CR can be used to compare the exposure levels of different hazard factors or different positions. CR > 1 indicates that the exposure to a hazard factor exceeds the OEL for that factor.

#### Quantitative analysis

##### Comparison of RRs among the six OHRA methods

The statistical differences among the RRs evaluated by the six OHRA methods reflect the differences among the evaluation results of the OHRA methods for the same occupational hazard factors.

##### Correlations among the RRs of the six OHRA methods

The correlations among the RRs obtained by the six methods were statistically analyzed.

##### Verification of relative accuracy of six OHRA methods

(1) The relative accuracy of the OHRA results obtained using the six OHRA methods in different industries was verified by comparing the consistency in RR values for different industries and inherent risks (IR) levels. Refer to Section “Description of typical industries,” for the classification of inherent risks in each industry.
(2) The relative accuracy of the evaluation result of each method was verified by evaluating the consistency in the RRs for different chemical toxicants and IRs. We selected four chemical toxicants (manganese and inorganic compounds, benzene, xylene, and ethyl acetate) to evaluate the accuracy of each method. The IR of a hazard factor depends on its inherent hazardous consequences and exposure probability. The IR increases as the inherent hazardous consequences become more severe and as the exposure concentration increases. In this study, the inherent hazardous consequences of a hazard factor were determined based on the RfC value of the EPA method. A larger RfC indicates less severe inherent hazard consequences. Table 3 shows the RfC values and exposure concentrations for each hazard factor. The IR values of the four hazard factors in the five industries decreases in the following order: IR_manganese_ > IR_benzene_ ≈ IR_xylene_ > IR_ethylacetate_.

**Table 3:** Quantitative comparison of RRs among the six OHRA models for four hazard factors.

**Hazard factors**		**Manganese and** **inorganic compounds**	**Benzene**	**Xylene**	**Ethyl acetate**
RfC (μg/m^3^)		0.05	30	100	3,500
CR [median (range)]		0.21 (0.06–0.62)^a,b,c^	0.05 (0.01–0.05)^a^	0.04 (0.019–0.66)^a^	0.000 (0.000–0.0004)
*n*		234	56	56	56
EPA	Risk level (range)	5	2–5	2–5	1–1.15
	RR [median (range)]	1.0 (1.0–1.0)^a,b,c^	0.4 (0.4–0.4)^a,b^	1.0 (1.0–1.0)^a^	0.2 (0.2–0.2)
COSHH	Risk level (range)	2	5	2	2
	RR [median (range)]	0.4^c^	1.0^a,b^	0.4	0.4
Singaporean	Risk level (range)	2–4	2–3	1–3	1–3
	RR [median (range)]	0.6 (0.4–0.6)^a,b,c^	0.4 (0.4–0.4)^a,b^	0.2 (0.2–0.6)^a^	0.2 (0.2–0.2)
ICMM	Risk level (range)	3–5	4–5	2–5	1–5
	RR [median (range)]	0.8 (0.6–1.0)^a,b^	0.8 (0.8–0.8)^a,b^	0.4 (0.4–0.8)^a^	0.2 (0.2–0.2)
Australian	Risk level (range)	2–3	3	1.7–2	2
	RR [median (range)]	0.6 (0.4–0.6)^a,b,c^	0.6^a,b^	0.4 (0.4–0.4)	0.4
Romanian	Risk level (range)	3–6	4–6	3–4	1
	RR [median (range)]	0.4 (0.3–0.4)^a,b,c^	0.4 (0.4–0.4)^a,b^	0.3 (0.3–0.3)^a^	0.1

RfC, reference concentration for inhalation toxicity; CR, concentration ratio; n, number of risk levels or RRs for each hazard factor; RR, risk ratio.

a P < 0.05 compared with petrol station;

b P < 0.05 compared with equipment repair;

c P < 0.05 compared with ship repair.

### Statistical analysis

The Kruskala–Wallis H(K) method was used to analyze the RRs and CRs of multiple independent samples. The Mann–Whitney *U* method was used to compare the RR or CR between two independent samples. The correlations between RR values were analyzed by Spearman correlation analysis (abnormal distribution).

## Results and discussion

### Comparison of RRs among the six OHRA methods

As shown in Table 4, among the six models, the highest RR was obtained by the EPA model [1.0 (0.4–1.0)] followed by the COSHH model [0.8 (0.4–1.0)], the ICMM model [0.8 (0.4–1.0)], the Australian model [0.6 (0.4–0.6)], the Singaporean model [0.4 (0.4–0.6)], and the Romanian model [0.4 (0.3–0.4)]. Thus, the RRs of the six methods decreased in the following order: RR_EPA_ > RR_COSHH_ > RR_ICMM_ > RR_Australian_ > RR_Singaporean_ > RR_Romanian_ (*P* < 0.05). This order is similar but not the same as the previously reported order: (22) RR_EPA_ > RR_COSHH_ > RR_Singaporean_ > RR_Australian_ > RR_ICMM_ and RR_Romanian_ (*P* < 0.05).

**Table 4:** Quantitative comparison of RRs among the six OHRA models in five industries.

**Industry**		**Mining of soil** **and sand**	**Ferrous casting**	**Ship repair**	**Equipment repair**	**Petrol station**	**Sum**
IR		Severe	Severe	Medium	Medium	Low	/
*n*		148	97	989	85	200	1,519
EPA	Risk level (range)	/	/	1–5	1–5	/	1–5
	RR [median (range)]	/	/	1.0 (0.4–1.0)^b^	1.0 (0.2–1.0)	/	1.0 (0.4–1.0)^e,f,g,h,i^
COSHH	Risk level (range)	5	2–5	2–4	2–5	5	2–5
	RR [median (range)]	1.0 (1.0–1.0)^b,c,d^	1.0 (1.0–1.0)^a,b,c^	0.4 (0.4–0.8)^a,b^	0.4 (0.4–0.8)^a^	1.0 (1.0–1.0)	0.8 (0.4–1.0)^e,f,g,h^
Singaporean	Risk level (range)	3–5	2–5	1–4	1–3	2	1–4
	RR [median (range)]	0.8 (0.8–0.8)^a,b,c^	0.8 (0.6–0.8)^a,b,c^	0.4 (0.4–0.6)^a^	0.4 (0.4–0.6)^a^	0.4 (0.4–0.4)	0.4 (0.4–0.6)^e,f,g^
ICMM	Risk level (range)	5	4–5	2–5	1–5	1–2	1–5
	RR [median (range)]	1.0 (1.0–1.0)^a,b,c,d^	1.0 (1.0–1.0)^a,b,c^	0.8 (0.6–1.0)^a^	1.0 (0.6–1.0)^a^	0.3 (0.2–0.4)	0.8 (0.4–1.0)^e,f^
Australian	Risk level (range)	3–4	2–4	2–4	2–3	2	2–4
	RR [median (range)]	0.6 (0.6–0.8)^a,b,c^	0.6 (0.6–0.6)^a,b,c^	0.6 (0.4–0.6)^a,b^	0.4 (0.4–0.6)^a^	0.4 (0.4–0.4)	0.6 (0.4–0.6)^e^
Romanian	Risk level (range)	4–6	3–6	3–6	1–6	1	1–6
	RR [median (range)]	0.4 (0.4–0.6)^a,b,c,d^	0.4 (0.4–0.4)^a,b,c^	0.4 (0.3–0.4)^a,b^	0.4 (0.3–0.4)^a^	0.1 (0.1–0.1)	0.4 (0.3–0.4)

IR, inherent risk according to the “Management catalog of occupational hazard risk classification for construction projects” issued by the State Administration of Work Safety of China (2012 edition); n, the number of risk levels or RRs for all hazard factors in each industry; RR, risk ratio.

a P < 0.05 compared with petrol station;

b P < 0.05 compared with equipment repair;

c P < 0.05 compared with ship repair;

d P < 0.05 compared with ferrous casting;

e P < 0.05 compared with the Romanian model;

f P < 0.05 compared with the Australian model;

g P < 0.05 compared with the ICMM model;

h P < 0.05 compared with the Singaporean model,

i P < 0.05 compared with the COSHH model.

The above results show that using different methods to evaluate the same risk produces different risk assessments, and the EPA and COSHH models result in the highest RR values. This may be because the EPA, Singaporean, and COSHH models are relatively objective, while the Australian, ICMM, and Romanian models are more subjective because they rely on the professional knowledge and work experience of evaluators. The EPA model produces a high RR because it evaluates risk using an order of magnitude formula (HQ = EC/RfC). The COSHH model does not consider the field exposure concentration, and the exposure concentration of each hazard factor in this study was less than the standard (CR < 1), resulting in a relatively high RR for this method. The Australian, ICMM, and Romanian methods rely on the experience and subjective judgment of an evaluator along with accurate accident occurrence data. However, the Romanian model has a more detailed rating (seven levels), which may explain why its evaluation results were relatively low (Tables 3, 4).

The results show that the different OHRA methods produce different risk assessment results for the same risk. Among the six OHRA methods, the EPA model is the most sensitive and produces the highest RR values, while the Romanian model results in the lowest RR values.

### Correlations among the RRs of the six OHRA methods

Table 5 shows the correlations among the RRs of the six OHRA methods. The RR of the COSHH model was not correlated with those of the ICMM and Romanian models, while correlations were found among the RRs of the other methods. Only the RR of the Singaporean model was positively correlated with those of the other five methods (*P* < 0.01), and the correlation coefficients were relatively greater and positive value. The RRs of the ICMM, Romanian, and Australian models were all positively correlated with each other. In a previous study, we found that the RR of the EPA model was not correlated with those of the COSHH, Romanian, and Australian models, while it was correlated with the RR of the ICMM model; meanwhile, the RR of the Singaporean model was positively correlated with those of the other five methods (*P* < 0.01) (22).

**Table 5:** Correlations among the RR values of the six OHRA methods.

	**RR_EPA_**	**RR_COSHH_**	**RR_Singaporean_**	**RR_ICMM_**	**RR_Australian_**	**RR_Romanian_**
RR_EPA_	1.000	–	–	–	–	–
RR_COSHH_	−0.355^*^	1.000	–	–	–	–
RR_Singaporean_	0.633^*^	0.125^*^	1.000	–	–	–
RR_ICMM_	0.442^*^	0.010	0.750^*^	1.000	–	–
RR_Australian_	0.472^*^	−0.152^*^	0.719^*^	0.815^*^	1.000	–
RR_Romanian_	0.252^*^	−0.043	0.696^*^	0.806^*^	0.935^*^	1.000
CR	0.174^*^	−0.023	0.506^*^	0.348^*^	0.443^*^	0.509^*^

* P < 0.001.

The RRs of the COSHH and EPA models were weakly correlated with those of the other methods, while the RR of the Singaporean model was positively correlated with those of the other five methods (*P* < 0.01). The EPA and COSHH models assess the hazard consequences of hazard factors based on their own unique parameters of hazard factors. The EPA model is based on IUR and RfC, while the COSHH model is based on risk-phrase. However, as a semi-quantitative method, the Singaporean model has characteristics of both quantitative and qualitative methods, resulting in good RR correlations with the other methods. The Romanian, Australian, and ICMM models are strongly influenced by the evaluator; thus, the differences among the results of these three methods could be reduced if the same evaluator applied these methods at the same time to evaluate the risk.

Since each OHRA method has its principle and methodology, the evaluation results of the methods are not necessarily correlated.

### Verification of relative accuracy of six OHRA methods in different industries

**Figure 1:**
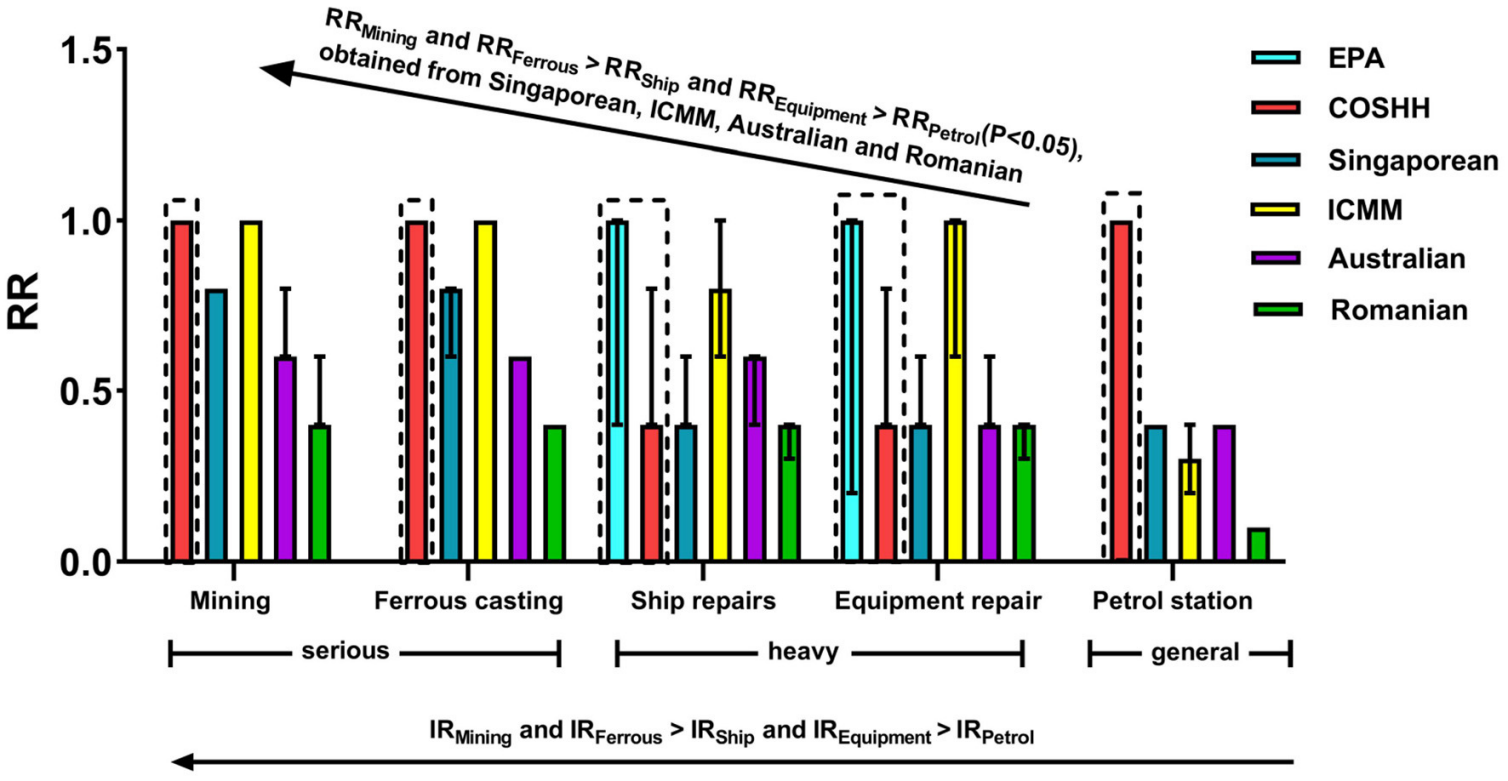
Quantitative comparison of RRs obtained for the five industries using the six models [median (interquartile spacing)]. The orders of RRs obtained using the Singaporean, ICMM, Australian, and Romanian models in the five industries were consistent with the orders of the three inherent risk levels in those industries. Only the Romanian model could distinguished the RRs of the five industries.

Figure 1 and Table 4 quantitatively compare the RRs among the six OHRA methods in the five industries. The EPA model could only assess risk in the ship repairs and equipment repair industries due to the lack of RfC values in the other industries. The sequence of RRs obtained from the Singaporean, ICMM, Australian, and Romanian models in the five industries was consistent with the sequence of the three inherent risk levels in those industries (*P* < 0.05), while the sequences were not consistent for the COSHH model. Only the Romanian model could distinguish the RR values of the five industries.

Most methods could distinguish differences among the industries with different inherent risk levels. This is inconsistent with our previous report in which the sequences of RRs obtained for five industries (leather, wood furniture, printing and dying of cloth or textile, printing on paper, and garment manufacturing) were consistent with the sequence of IR only for the EPA, Singaporean, and COSHH models (22).

The exposure concentration was used to determine the occurrence probability in the EPA, Singaporean, ICMM, Australian, and Romanian models. In contrast, the amount of hazard factor (ML-L-T) was used in the COSHH model, which was more rough than other methods. Compared with previous studies (22) (5,000 employees from 10 enterprises), the results of this study were more representative due to the larger amount of data (16,000 people from 151 enterprises). According to a report on surveillance and OHRA for key occupational diseases in Zhejiang province from 2010 to 2020, among 59 manufacturing sectors, soil sand mining and ferrous metal casting ranked second and fifth in risk level, respectively, while ship repair, equipment repair, and gasoline stations ranked 12th, 38th, and 57th, respectively. This further confirms that most OHRA methods can distinguish differences among industries with different IRs; however, the ICMM, Australian, and Romanian models should be applied simultaneously by the same evaluator.

In the present study, only the Romanian model could distinguish the RRs of the five industries (Table 3). This might be because the assessment results of the Romanian model are divided into seven grades, compared with four or five grades for the other five methods. As shown in Table 3, the EPA, Singaporean, and Romanian models distinguished the RRs of the four hazard factors.

### Verification of relative accuracy of the six OHRA methods for different chemical toxicants

**Figure 2:**
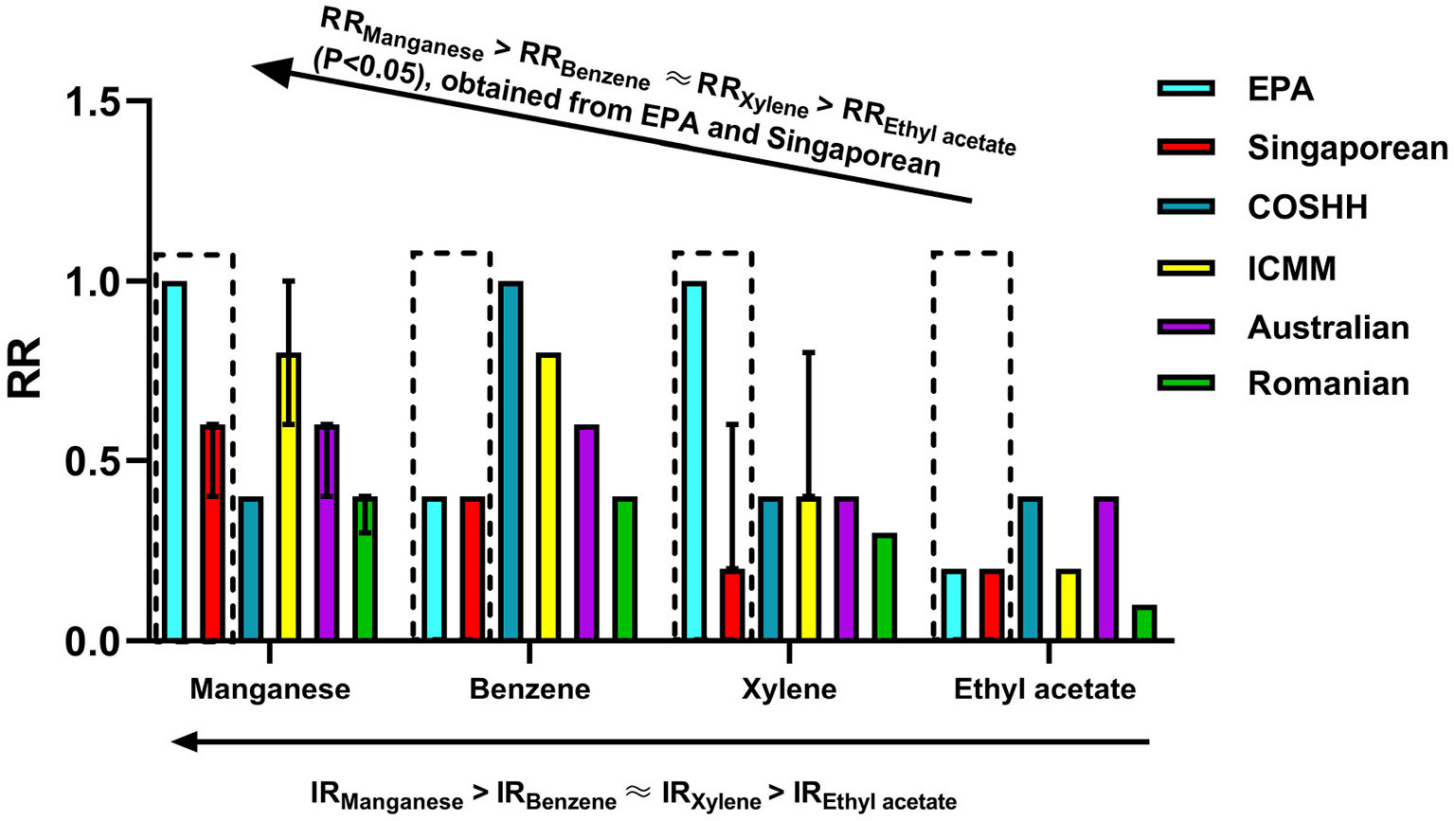
Quantitative comparison of the RRs obtained for four hazard factors (manganese and inorganic compounds, benzene, xylene, and ethyl acetate) using the six methods [median (interquartile spacing)]. The IR order of the four hazard factors in the five industries was: IR_manganese_ > IR_benzene_ ≈ IR_xylene_ > IR_ethylacetate_. The EPA and Singaporean models effectively distinguished the inherent risks (IRs) among the four hazard factors using the RRs (*P* < 0.05). According to the EPA method, the RRs order of the four hazard factors was: RR_manganese_ > RR_xylene_ > RR_benzene_ > RR_ethylacetate_ (*P* < 0.05), while that for the Singaporean method was: RR_manganese_ > RR_benzene_ > RR_xylene_ > RR_ethylacetate_ (*P* < 0.05).

Figure 2 and Table 3 quantitatively compares the RRs obtained using the six OHRA methods for the four hazard factors (manganese and inorganic compounds, benzene, xylene, and ethyl acetate). The IR values decreased in the following order: IR_manganese_ > IR_benzene_ ≈ IR_xylene_ > IR_ethylacetate_. The EPA and Singaporean models effectively distinguished the IR values among the four hazard factors (manganese and inorganic compounds, benzene, xylene, and ethyl acetate) using the RRs (*P* < 0.05). According to the EPA model, the sequence of RRs for the four hazard factors at work was RR_manganese_ > RR_xylene_ > RR_benzene_ > RR_ethylacetate_ (*P* < 0.05), while that for the Singaporean model was RR_manganese_ > RR_benzene_ > RR_xylene_ > RR_ethylacetate_ (*P* < 0.05)_._ Thus, the EPA and Singaporean models were highly accurate for assessing the inherent risks of chemical toxicants, in agreement with our past findings. We previously found that only the EPA and Singaporean models can effectively distinguish the IR values of xylene and ethyl acetate from the painting process. This may be related to the poor ability of the other four qualitative methods, which do not directly consider on-site exposure concentration, to assess exposure (22).

In this study, the RR values for xylene and benzene estimated by the EPA model were opposite order to those obtained by the Singapore model. The IR values of xylene and benzene depend on their inherent hazard consequences and exposure concentrations. The non-carcinogenic hazard posed by benzene is more severe than that of xylene [RfC_xylene_ (100 μg/m^3^) > RfC_benzene_ (30 μg/m^3^)], while there is no significant difference between CR_benzene_ [0.05 (0.01–0.05)] and CR_xylene_ [0.04 (0.019–0.66)] (*P* > 0.05). According to the risk definition, the non-carcinogenic risk of benzene is slightly higher than that of xylene; however, based on the EPA model, the non-carcinogenic risk of xylene is greater than that of benzene. This discrepancy may be because the EPA model does not assess the health risks of chemical toxicants by simply comparing hazard consequences and exposure concentrations; rather, the EPA model uses the following quantitative assessment formula: HQ = EC × 1,000/RfC. Although the statistical analysis [the Kruskala–Wallis H(K) method] failed to distinguish between the exposure concentrations of benzene and xylene, the EPA model could distinguish risk differences between benzene and xylene, which gave a more accurate assessment of the difference in risk, and the results of the EPA model were completely contrary to those obtained by the Singapore model.

Based on the above results, the EPA model is relatively more accurate and sensitive than the Singaporean model in assessing chemical toxicants, especially for those with carcinogenic properties. This conclusion applies only to dust-free chemical poisons and is based on the inherent risk of identifying risk factors at on-site exposure concentrations.

## Conclusions

The following conclusions can be drawn based on the findings of this study.

(1) The use of different OHRA methods for the same risk produced different results. Among the six OHRA methods, the EPA model was the most sensitive and produced the highest RR values, whereas the Romanian model resulted in the lowest RR values. Thus, it is necessary to select the appropriate method based on the specific risks and working environments.
(2) Among the OHRA methods, the Singaporean model had the strongest RR correlation with the other methods (*P* < 0.01).
(3) Among the six methods, the EPA model had the relatively highest accuracy in assessing chemical toxicants, followed by the Singaporean model. This conclusion applies only to dust-free chemical poisons and is based on the inherent risk of identifying risk factors at on-site exposure concentrations.
(4) Compared to the other methods, the EPA and Romanian models better differentiated toxicity risk.

Further research is needed in this field. For example, more quantitative comparison methods are needed to explore the advantages and application fields (e.g., comparison of the risks of percutaneous absorbed substances, poisons with and without on-site concentrations, and enterprises of different sizes) of each OHRA method to provide a scientific basis for the OHRA of chemical toxicants in workplaces.

## Data availability statement

The raw data supporting the conclusions of this article will be made available by the authors, without undue reservation.

## Author contributions

QX studied the design, collection, analysis of data, interpretation of data, and gave final approval of the manuscript. YC, MZ, HZ, ZZ, LX, WY, HR, PW, and XS contributed to study design, collection, detection, analysis and field investigation, and manuscript writing. All authors contributed to the article and approved the submitted version.

## Funding

This work was supported by the Zhejiang Provincial Foundation Public Welfare Research Project (No. LGC21H260001), Zhejiang Health Science and Technology Plan (Nos. 2021KY616, 2022ZH030, 2021KY613, and 2022RC120), and Project of South Zhejiang Institute of Radiation Medicine and Nuclear Technology (No. ZFY-2021-K-003).

## Conflict of interest

Author LX was employed by Zhejiang Tianlan Environmental Protection Engineering Co., Ltd. Author XS was employed by Zhejiang Jidi Testing Technology Co., Ltd. The remaining authors declare that the research was conducted in the absence of any commercial or financial relationships that could be construed as a potential conflict of interest.

## Publisher's note

All claims expressed in this article are solely those of the authors and do not necessarily represent those of their affiliated organizations, or those of the publisher, the editors and the reviewers. Any product that may be evaluated in this article, or claim that may be made by its manufacturer, is not guaranteed or endorsed by the publisher.
